# Changes in Cardiac-Arterial Coupling After Cardiac Surgery for Aortic Valve Disease

**DOI:** 10.31083/RCM49206

**Published:** 2026-06-08

**Authors:** Anna Giani, Oscar Plunde, Cristina Oliveira da Silva, Claudia Martins Sequeira, Kambiz Shahgaldi, Luis Miguel Cardoso Dos Santos, Francesco Fantin, Mauro Zamboni, Aristomenis Manouras, Anders Franco-Cereceda, Magnus Bäck

**Affiliations:** ^1^Department of Medicine Solna, Karolinska Institutet, 17176 Stockholm, Sweden; ^2^Department of Medicine, Section of Geriatrics, University of Verona, 37126 Verona, Italy; ^3^Heart and Vascular Center, Karolinska University Hospital, 17176 Stockholm, Sweden; ^4^Department of Cardiology, Danderyd Hospital, 18288 Stockholm, Sweden; ^5^Department of Clinical Physiology, Danderyd Hospital, 18288 Stockholm, Sweden; ^6^Centre for Medical Sciences—CISMed, Department of Psychology and Cognitive Science—DIPSCO, Section of Geriatric Medicine, University of Trento, 38068 Rovereto, Italy; ^7^Department of Molecular Medicine and Surgery, Karolinska Institutet, 17176 Stockholm, Sweden; ^8^Centre Hospitalier Régional Universitaire de Nancy, 54500 Vandoeuvre-lès-Nancy, France; ^9^Université de Lorraine, Inserm, DCAC, F-54000 Nancy, France

**Keywords:** ventricular-arterial coupling, atrial-arterial coupling, arterial stiffness, aortic valve replacement, atrial function, ventricular function

## Abstract

**Background:**

Aortic valve disease affects vascular and cardiac remodeling and function, with changes observed after cardiac surgery. Ventricular-arterial coupling (VAC), which reflects the interaction between the left ventricle and the arterial system, may be altered by aortic valve replacement (AVR). Therefore, this study aimed to determine changes in VAC and atrial-arterial coupling (AAC) in patients with aortic valve disease before and after AVR.

**Methods:**

A total of 41 patients (median age 67 years, interquartile range (IQR) 62–71) undergoing AVR, including 24 (58.5%) with aortic stenosis (AS), were evaluated before and early (3 days) after cardiac surgery. Left atrial reservoir strain (LARS) and left ventricular (LV) global longitudinal strain (GLS) were assessed by echocardiography. Carotid–femoral pulse wave velocity (cfPWV), brachial-ankle-PWV (baPWV), and cardio-ankle vascular index (CAVI) were measured. VAC and AAC were defined as ratios of arterial stiffness to cardiac strain (CAVI/GLS, CAVI/LARS, baPWV/GLS, baPWV/LARS, cfPWV/GLS, cfPWV/LARS).

**Results:**

Atrial and ventricular strain and arterial stiffness significantly worsened after surgery. Ventricular-arterial uncoupling was reflected by a substantial increase in arterial stiffness to LV strain ratios (*p* < 0.001 for each). Moreover, AAC, measured as CAVI/LARS or PWV/LARS, significantly increased after surgery, with *p* < 0.001 for CAVI/LARS and baPWV/GLS, and *p* = 0.035 for cfPWV/LARS.

**Conclusions:**

Worsened ventricular-arterial uncoupling can be detected early after cardiac surgery using novel measures of VAC, including ratios of peripheral arterial stiffness to cardiac deformation. This study extends these findings by demonstrating atrial-arterial uncoupling, suggesting a generalized cardiac-arterial uncoupling after valve surgery.

## 1. Introduction

Aortic valve disease affects valve function and vascular and cardiac remodeling, involving the left atrium (LA), left ventricle (LV), and arterial stiffening. Ventricular-arterial coupling (VAC) can affect patient outcomes in several conditions and potentially even after aortic valve replacement (AVR). However, the interaction between vascular remodeling and upstream atrial function in aortic stenosis has not yet been explored.

Arterial stiffness reflects a complex network of inflammatory and atherogenic pathways that lead to elevated arterial blood pressure (BP) [1]. Pressure overload drives cardiac remodeling, characterized by LV wall thickening and LA dilation. Carotid–femoral pulse-wave velocity (cfPWV), obtained by applanation tonometry, is the gold-standard technique for assessing arterial stiffness [2] and is a reliable predictor of mortality risk across different patient populations. However, cardio-ankle vascular index (CAVI) and the derived brachial-ankle PWV (baPWV) can assess arterial stiffness across a larger proportion of the arterial tree, and are less dependent on BP at the time of measurement [3], while still maintaining significant associations with cardiovascular events and disorders.

Echocardiographic evaluation of LV global longitudinal strain (GLS) and LA reservoir strain (LARS) provides sensitive markers of subtle changes in longitudinal LV and LA function [4].

Notably, although cardiac and vascular parameters each have strong and independent prognostic value, a simultaneous assessment of VAC could better capture potential mismatches in the interaction between the LV and the arterial system, thereby providing a broader perspective on this complex phenomenon. While a gold-standard technique has yet to be established [5], VAC can be estimated noninvasively by echocardiographic measurement of the ratio of effective arterial elastance (Ea) to LV end-systolic elastance (Ees) [5]. Recently, several studies [6,7] have highlighted the role of the arterial stiffness-to-ventricular strain ratio, which may even be superior to the traditional echocardiographic VAC calculation [8].

Therefore, this study aimed to investigate VAC and atrial-arterial coupling (AAC) in patients with aortic valve disease before and after AVR.

## 2. Materials and Methods

This study retrospectively analyzed echocardiographic examinations from patients in the DAVAACA study who underwent AVR for either aortic stenosis (AS) or aortic regurgitation (AR) at Karolinska University Hospital (Stockholm, Sweden). A total of 88 patients from the DAVAACA study with available arterial stiffness measurements, as previously described [9], were initially considered. After excluding 47 patients with incomplete echocardiographic data, 41 patients remained for analysis. Detailed patient selection procedures have been reported previously [9]; a flowchart is shown in **Supplementary Fig. 1**. All participants provided written informed consent. The study was approved by the local ethics committee (2012/1633-31/4, amendment 2016/2346-32) and conducted in accordance with the Declaration of Helsinki.

Clinical and anthropometric data were recorded. The closest available echocardiography studies in the electronic health record before and after surgery were selected; the analysis was performed offline using vendor-independent software (Version 3.0, Tomtec-TTA2, Tomtec Imaging Systems GmbH, 2019, Unterschleissheim, Germany).

LV GLS was assessed using an 18-segment model (6 segments in each of the 3 apical views) and calculated as the average of all segments [4]. For the LA, appropriate LA strain software was applied to the apical four- and two-chamber views; the results were averaged, with LARS defined as the peak systolic strain [4].

All arterial stiffness measurements were obtained the day before surgery; postoperative measurements were performed 3 days after surgery. CAVI, right brachial BP, and heart rate (HR) were measured, and mean arterial pressure (MAP) and pulse pressure (PP) were calculated using the VaSera-1500 device (Fukuda, Denshi). CAVI is based on the β-stiﬀness index and the Bramwell–Hill Formula [9]; baPWV was estimated using the formula (0.5934 × height (cm) + 14.4724)/tba. The cfPWV was measured by applanation tonometry (Sphygmocor, AtCor Medical, Sydney, Australia); a detailed description of the calculation method has been provided previously [9].

VAC was calculated as stiffness-to-strain ratios, namely: cfPWV/GLS, CAVI/GLS, and baPWV/GLS. AAC was obtained as cfPWV/LARS, CAVI/LARS, and baPWV/LARS. GLS was considered as an absolute value (|GLS|).

Continuous variables are presented as medians with interquartile ranges (IQRs) and categorical variables as proportions. Baseline characteristics of patients with and without AS were compared using the Mann–Whitney test; the same test was performed to compare the post-pre-surgery deltas between patients with and without AS. Baseline and post-surgery characteristics were compared using the Wilcoxon signed-rank test. Multiple linear regression was performed to identify potential predictors of VAC and AAC; non-normally distributed dependent variables were log-transformed before analysis. Statistical significance was set at *p* < 0.05. All analyses were performed using R (https://www.r-project.org, version 4.1.2, 2021) and SPSS 23.0 (IBM, Armonk, NY, USA).

## 3. Results

A total of 41 patients were included, with a median age of 67 years (IQR 62–71), and 28 (68%) were male. The main hemodynamic, echocardiographic, and stiffness characteristics are reported in Table 1.

**Table 1.** **Baseline hemodynamic, echocardiographic, and arterial stiffness characteristics of the study population were compared between patients with and without AS, and in the overall population before and after surgery**.

	Overall (n = 41)		AS (n = 24)		Not AS (n = 17)			Before (overall)		After (overall)		
	Median	IQR	Median	IQR	Median	IQR	*p*-value	Median	IQR	Median	IQR	*p*-value
Age (years)	67.0	62.0–71.0	67.5	63.5–72.3	67.0	59.0–71.0	0.633					
Male sex (n, %)	28	68.3	15	62.5	13	76.5	0.274					
GLS (%)	–16.1	–17.4 to –14.4	–16.1	–17.5 to –14.7	–16.0	–17.1 to –14.4	0.654	–16.1	–17.4 to –14.4	–11.7	–13.7 to –9.65	<0.001
LARS (%)	30.8	25.9–32.7	31.3	27.7–32.7	29.7	25.0–32.6	0.489	30.8	25.9–32.7	24.7	19.5–29.3	<0.001
EF (%)	53.9	50.3–61.4	58.1	50.5–62.2	53.2	50.0–55.6	0.326	53.9	50.3–61.4	52.5	46.6–57.8	0.245
LAVi (mL/m^2^)	30.5	26.3–39.2	30.5	26.9–37.8	30.4	25.7–40.0	0.877	30.5	26.3–39.2	32.1	27.6–37.8	0.863
SBP (mmHg)	144	133–153	137	132–148	152	140–159	0.004	144	133–153	132	119–143	0.040
DBP (mmHg)	84.0	76.5–88.0	84.5	83.0–89.4	79.0	74.0–87.0	0.152	84.0	76.5–88.0	77.5	69.3–85.0	0.022
MAP (mmHg)	102	98.0–110	102	99.0–106	102	98.0–114	0.496	102	98.0–110	96.0	87.0–103	0.010
PP (mmHg)	58.0	48.5–69.5	51.5	45.8–57.5	66.0	60.0–77.0	<0.001	58.0	48.5–69.5	53.0	47.0–64.8	0.226
HR (bpm)	65.0	57.5–71.0	69.0	61.0–76.8	59.0	57.0–67.0	0.067	65.0	57.5–71.0	83.0	73.8–89.5	<0.001
cfPWV (m/s)	7.80	6.70–8.65	7.47	6.85–8.10	8.30	6.10–9.70	0.269	7.80	6.70–8.65	8.10	6.70–8.93	0.898
baPWV (cm/s)	1318	1199–1458	1283	1195–1357	1402	1213–1575	0.127	1318	1199–1458	1461	1225–1586	0.022
CAVI	7.55	6.81–8.46	7.63	6.95–8.17	7.43	6.40–8.94	0.600	7.55	6.81–8.46	9.04	7.88–9.53	<0.001
ABI	1.18	1.12–1.25	1.16	1.11–1.20	1.25	1.12–1.28	0.109	1.18	1.12–1.25	1.15	1.06–1.23	0.032
CAVI/|GLS|	0.48	0.40–0.59	0.47	0.40–0.59	0.52	0.40–0.65	0.577	0.48	0.40–0.59	0.73	0.66–0.93	<0.001
CAVI/LARS	0.25	0.21–0.30	0.25	0.22–0.28	0.26	0.21–0.32	0.514	0.25	0.21–0.30	0.37	0.28–0.46	<0.001
baPWV/|GLS|	88.4	71.8–104	78.0	70.0–103	94.2	76.7–113	0.338	88.4	71.8–104	123	111–146	<0.001
baPWV/LARS	45.4	39.2–52.7	43.6	38.5–49.2	46.2	39.5–62.2	0.290	45.4	39.2–52.7	59.2	46.5–73.2	<0.001
cfPWV/|GLS|	0.51	0.39–0.65	0.46	0.42–0.55	0.58	0.37–0.75	0.483	0.51	0.39–0.65	0.69	0.52–0.81	<0.001
cfPWV/LARS	0.25	0.21–0.32	0.25	0.21–0.31	0.27	0.21–0.36	0.421	0.25	0.21–0.32	0.33	0.23–0.40	0.035

In stiffness-to-strain ratios, the absolute value of GLS is considered. IQR, interquartile range; ABI, ankle-brachial index; AS, aortic stenosis; AVR, aortic valve replacement; baPWV, brachial-ankle pulse wave velocity; CAVI, cardio-ankle vascular index; cfPWV, carotid-femoral pulse wave velocity; DBP, diastolic blood pressure; EF, ejection fraction; HR, heart rate; MAP, mean arterial pressure; GLS, global longitudinal strain; LARS, left atrial reservoir strain; LAVi, left atrial volume index; PP, pulse pressure; SBP, systolic blood pressure.

Most patients had a diagnosis of AS at baseline (n = 24, 58.5%); the remaining patients had AR (n = 13, 31.7%) or ascending aortic dilation/aneurysm (n = 4, 9.8%). At baseline, patients with AS had lower systolic blood pressure and lower pulse pressure than those without AS (Table 1).

Comparing baseline and early post-surgery evaluations (Table 1) revealed that, in addition to changes in blood pressure and heart rate, the myocardial deformation indices GLS and LARS were significantly decreased (*p *< 0.001 for both). In contrast, postoperative arterial stiffness increased significantly for CAVI (*p* < 0.001) and showed a trend toward higher baPWV (*p* = 0.022), whereas cfPWV did not change significantly.

**Fig. 1.**
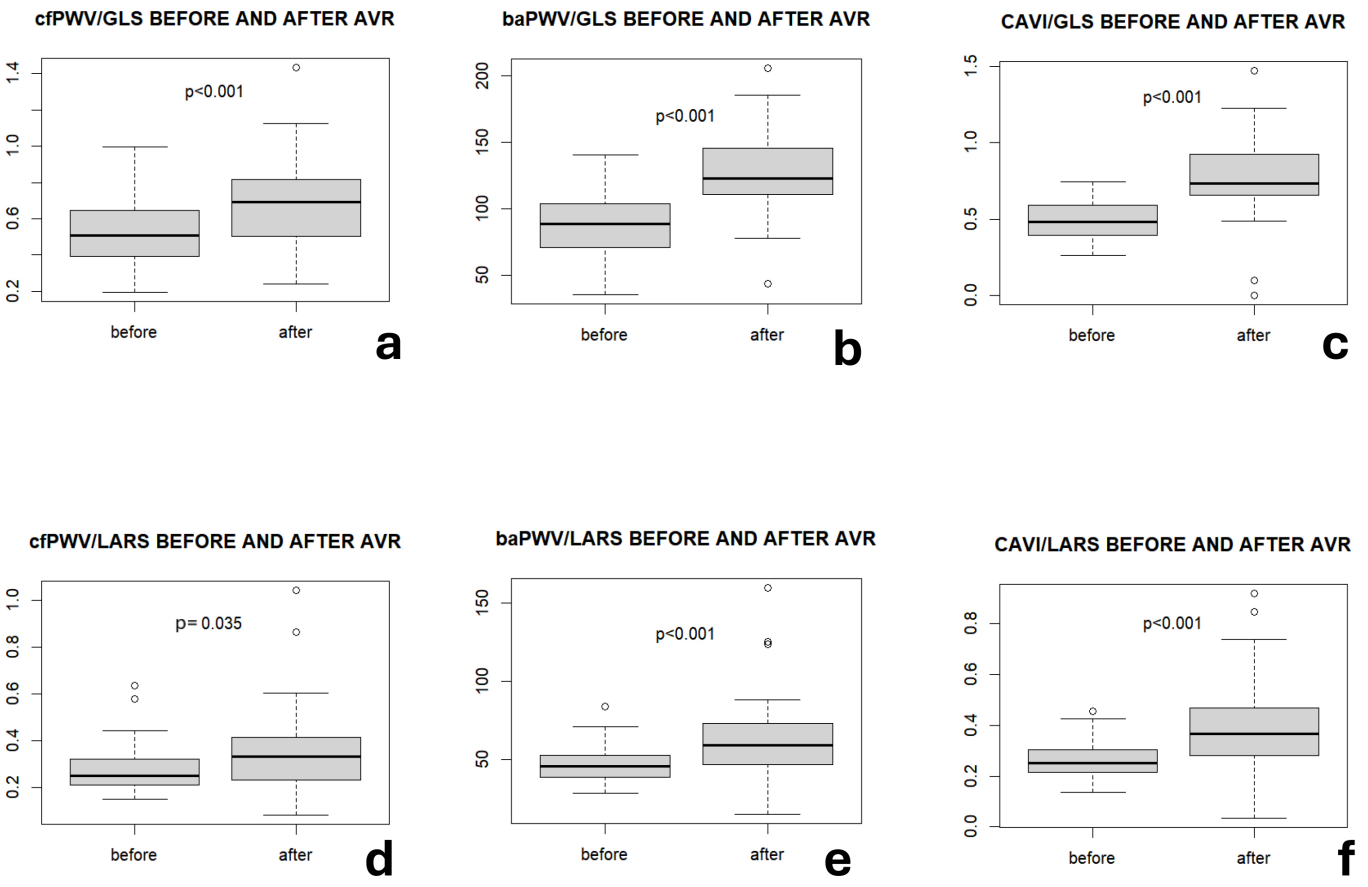
**Stiffness-to-strain ratios before and after surgery**. (a) cfPWV/GLS before and after surgery in the overall population. (b) baPWV/GLS before and after surgery in the overall population. (c) CAVI/GLS before and after surgery in the overall population. (d) cfPWV/LARS before and after surgery in the overall population. (e) baPWV/LARS before and after surgery in the overall population. (f) CAVI/LARS before and after surgery in the overall population. GLS was considered an absolute value. baPWV, brachial-ankle pulse wave velocity; CAVI, cardio-ankle vascular index; cfPWV, carotid-femoral pulse wave velocity; GLS, global longitudinal strain; LARS, left atrial reservoir strain.

VAC was defined as the ratio of arterial stiffness to ventricular strain, and showed a significant increase for each of the assessed indices: CAVI/GLS, baPWV/GLS (both *p* < 0.001), and cfPWV/GLS (*p* < 0.001; Fig. 1).

AAC also consistently increased after surgery, reflecting a disproportionate mismatch between atrial and arterial dynamics; cfPWV/LARS (*p* = 0.035), CAVI/LARS (*p *< 0.001), and baPWV/LARS (*p* < 0.001) ratios followed the same incremental trend (Table 1; Fig. 1).

The changes in atrial, ventricular, and arterial dynamics and stiffness were also analyzed, comparing patients with and without AS (Table 2). With regard to these parameters, no statistically significant difference was detected in pre- to postoperative changes between patients with and without AS, although a numerically larger increase was observed in patients without AS.

**Table 2.** **After-before variation (Δ) for the main characteristics of the study population, comparing patients with and without AS**.

	AS		Not AS		
	Median	IQR	Median	IQR	*p*-value
Δ|GLS| (%)	–3.94	–5.38 to –1.88	–4.38	–8.46 to –2.54	0.305
ΔLARS (%)	–4.04	–8.30–0.382	–4.69	–9.78–1.45	0.966
ΔEF (%)	–2.14	–7.23–3.92	–5.00	–8.45–2.46	0.485
ΔLAVi (mL/m^2^)	–1.42	–5.16–6.57	0.0615	–7.44–8.24	0.836
ΔSBP (mmHg)	4.00	–17.0–11.0	–15.0	–37.5 to –1.00	0.032
ΔDBP (mmHg)	–6.00	–14.5 to –1.00	–4.00	–12.5–7.00	0.123
ΔMAP (mmHg)	–5.00	–11.0–1.00	–5.00	–17.0–1.00	0.883
ΔPP (mmHg)	9.00	–5.00–17.0	–14.0	–31.0 to –7.00	<0.001
ΔHR (bpm)	16.0	8.00–21.5	19.0	7.00–32.0	0.342
ΔcfPWV (m/s)	0.01	–0.70–1.10	–0.10	–0.30–0.20	0.463
ΔbaPWV (cm/s)	130	–16.5–180	121	–109–194	0.363
ΔCAVI	1.26	0.932–1.52	1.34	0.720–1.96	0.955
ΔABI	–0.02	–0.12–0.083	–0.08	–0.15–0.06	0.327
ΔcfPWV/GLS	0.21	–0.01–0.30	0.20	0.17–0.35	0.361
ΔbaPWV/GLS	33.0	11.9–48.9	55.5	25.9–81.5	0.134
ΔCAVI/GLS	0.19	0.06–0.43	0.34	0.23–0.58	0.202
ΔcfPWV/LARS	0.07	–0.02–0.14	–0.01	–0.03–0.12	0.547
ΔbaPWV/LARS	12.4	–0.30–25.0	7.50	–0.11–20.2	0.780
ΔCAVI/LARS	0.09	0.03–0.16	0.06	0.03–0.19	0.825

GLS is considered in absolute value. ABI, ankle-brachial index; AS, aortic stenosis; AVR, aortic valve replacement; baPWV, brachial-ankle pulse wave velocity; CAVI, cardio-ankle vascular index; cfPWV, carotid-femoral pulse wave velocity; DBP, diastolic blood pressure; EF, ejection fraction; HR, heart rate; MAP, mean arterial pressure; GLS, global longitudinal strain; LARS, left atrial reservoir strain; LAVi, left atrial volume index; PP, pulse pressure; SBP, systolic blood pressure.

Multiple regression models were used to investigate potential predictors of VAC (**Supplementary Table 1**), including age, AS indication for surgery, baseline MAP, and HR as independent variables; before surgery, age and MAP were significant predictors of cfPWV/GLS, baPWV/GLS, and CAVI/GLS (borderline for age). The same variables, together with HR, were significant predictors of AAC assessed as cfPWV/LARS. When considering VAC after surgery, the AS indication for surgery was at the limit of significance as a predictor of CAVI/GLS (β = 0.233; SE = 0.113; *p* = 0.051).

## 4. Discussion

The major findings of this exploratory, single-center, retrospective study of 41 patients undergoing cardiac surgery indicate a general postoperative cardiac-arterial uncoupling. First, novel indices integrating LV strain and peripheral arterial stiffness, assessed by CAVI and PWV, detected ventricular-arterial uncoupling early after cardiac surgery. Second, this is the first indication of atrial-arterial uncoupling.

A general worsening of LA and LV function is evident early after surgery. Likewise, arterial stiffness indices increase after surgery, consistent with previous findings from the DAVAACA study [9], in which CAVI increased after AVR, particularly in patients with AS. Since arterial stiffness may be underestimated in AS, preoperative CAVI appears to be associated with AS severity [10], whereas postoperative stiﬀness may better reﬂect the true vascular status of the patient [11]. Notably, a weak, prolonged pulse (e.g., in AS) may contribute to lower CAVI values [11]. Although both GLS and vascular stiffness worsened after cardiac surgery, regardless of the surgical indication, combining vascular and ventricular function (i.e., CAVI/GLS) differentiated AS from non-AS surgery indications. Within the pathophysiological framework of AS, these findings suggest that ventricular-arterial uncoupling is less pronounced after relieving the LV of the AS load via AVR, as evidenced by the arterial component being better tolerated, with a smaller decrease in GLS.

As mentioned above, arterial stiffness increases after surgical procedures due to pressure loading following relief of the obstruction; however, correction of AS shifts part of the load borne by the LV to the aortic walls; in this context, arterial stiffness may play a pivotal role in influencing the VAC. This mechanism might also support the regression model results, identifying AS indication as a significant predictor of post-operative CAVI/GLS independently of the expected VAC predictors such as age, MAP, and HR.

Notably, atrial function and arterial stiffness also worsen concomitantly postoperatively and may contribute to generalized cardiovascular coupling, given the direct exposure of the LA chamber to LV pressures when the mitral valve is open [12], ultimately affecting both VAC and AAC.

Hence, the present study introduces novel measures of AAC (PWV/LARS and CAVI/LARS) that appear to worsen after surgery, with a similar trend regardless of the AVR indication. Previous evidence has demonstrated an inverse association between atrial function and arterial stiffness (cfPWV) [13,14]. Furthermore, studies in hypertensive patients have reported significant associations between left atrial volume and both cfPWV [15] and baPWV [16]. Therefore, the findings of this study broaden the perspective on cardiac-vascular coupling [14] by proposing a more comprehensive index that incorporates LA function, which, potentially passing through atrial-ventricular coupling [5], ultimately interacts with vascular function.

The link between vascular function and valvular heart disease, particularly AS, is underpinned by shared pathophysiological processes. For example, reduced arterial compliance in peripheral artery disease, reflected by an elevated ankle-brachial index (ABI), predicts the extent of aortic valve calcification [17].

Uncoupling is further supported by disproportionate deterioration in LV and LA function and by an increase in arterial stiffness (CAVI and PWV) after AVR. Thus, the results of the present study support the hypothesis that cardiac surgery leads to cardiac-vascular uncoupling, driven by changes in both atrial and LV function and arterial stiffness.

## 5. Limitations

However, several limitations should be acknowledged. First, the small sample size may have reduced the statistical power to detect some associations; further studies in larger populations are needed to confirm these findings. Second, although the presence of AF may influence LARs and, consequently, AAC, the limited sample size precluded the exclusion of patients with AF for a dedicated sensitivity analysis in this subpopulation. Third, the short interval between surgery and postoperative evaluations limits the assessment of reverse remodeling. Although functional improvement after AVR would be expected, the early timing of the postoperative evaluation may not allow for the full expression of compensatory mechanisms and structural remodeling. Further studies are warranted to compare stiffness-to-strain ratios with other conventional VAC measurements (e.g., Ea/Ees). Moreover, additional studies are needed to determine whether VAC and AAC serve as possible predictors of clinical outcomes.

## 6. Conclusions

The stiffness-to-strain ratio is emerging as a robust measure of VAC [6,7,8], with both CAVI [6] and PWV [7] previously applied. However, to our knowledge, this is the first report describing atrial-arterial changes after AVR and the contribution of these changes to postoperative cardiac-vascular uncoupling. Overall, these results provide an extensive characterization of VAC and highlight potential pathophysiological mechanisms linking arterial and cardiac dysfunction.

## Data Availability

The data that support the findings of this study are not publicly available. The study presented here has been subject to an application to an ethics board and approved for publication, in line with the specific aim of our research project. With reference to the European General Data Protection Regulation (GDPR), the data are personal data and thereby protected by secrecy.

## Abbreviations

GLS, global longitudinal strain; LARS, left atrial reservoir strain; EF, ejection fraction; LAVi, left atrial volume index; SBP, systolic blood pressure; DBP, diastolic blood pressure; MAP, mean arterial pressure; PP, pulse pressure; HR, heart rate; cfPWV, carotid-femoral pulse wave velocity; baPWV, brachial-ankle pulse wave velocity; CAVI, cardio-ankle vascular index.
