# Supplementary material for: Changes in Cardiac-Arterial Coupling After Cardiac Surgery for Aortic Valve Disease
Source: Rev Cardiovasc Med. 2026 Jun 8;27(6):49206. doi: 10.31083/RCM49206 (PMC13339173; doi:10.31083/RCM49206)
Supplement: Supplementary file 1 [file 2153-8174-27-6-49206-s1.zip › Supplementary Material.docx]

Supplementary Fig. 1. Flowchart of the study population selection


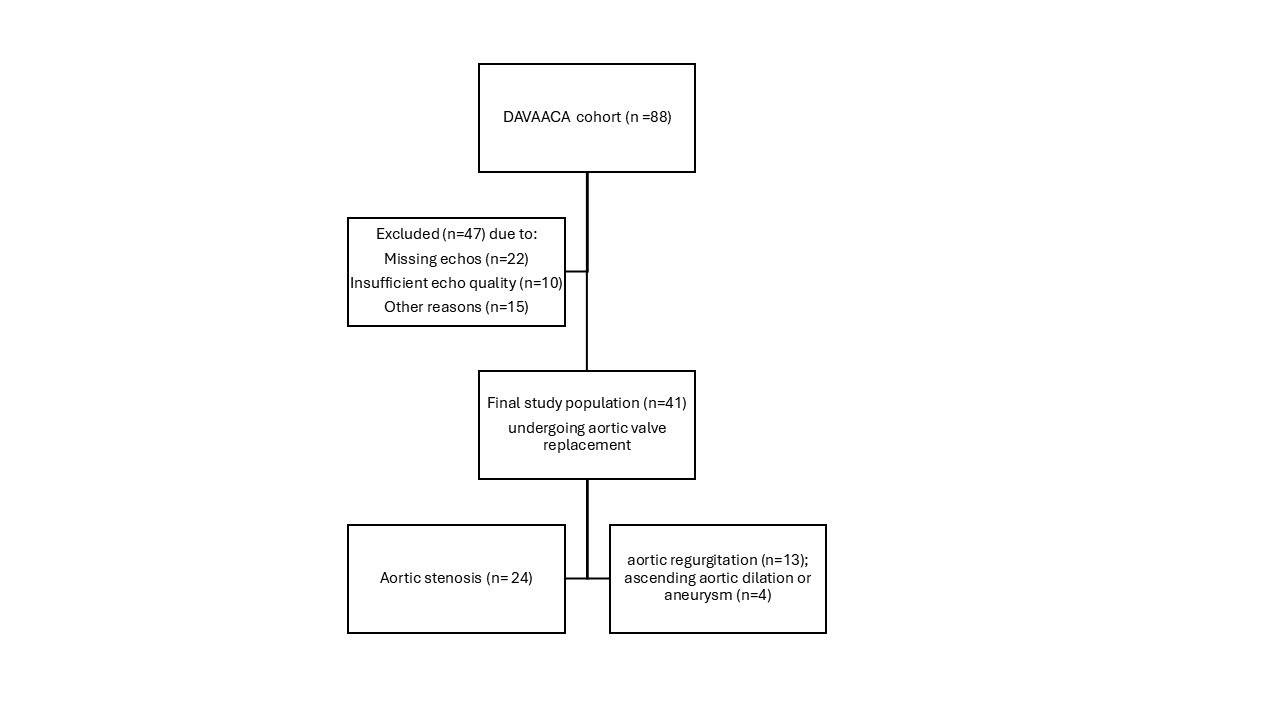


Supplementary Table 1: Regression models

| BEFORE AVR | | | | | | | AFTER AVR | | | | | | |
| --- | --- | --- | --- | --- | --- | --- | --- | --- | --- | --- | --- | --- | --- |
|  |  | **β** | **SE** | **t** | **p** | **R2** |  |  | **Β** | **SE** | **t** | **p** | **R2** |
| CAVI/GLS |  |  |  |  |  | 0.305 | CAVI/GLS |  |  |  |  |  | 0.229 |
|  | Intercept | -0.233 | 0.320 | -0.730 | 0.471 |  |  | Intercept | 172.357 | 0.740 | 2.329 | 0.030 |  |
|  | AS | 0.002 | 0.045 | 0.037 | 0.971 |  |  | AS | -0.233 | 0.113 | -2.071 | 0.051 |  |
|  | HR | -0.003 | 0.002 | -15.857 | 0.124 |  |  | HR | -5.65e−4 | 0.004 | -0.143 | 0.888 |  |
|  | MAP | 0.006 | 0.002 | 26.407 | 0.013 |  |  | MAP | -0.007 | 0.004 | -1.739 | 0.097 |  |
|  | Age | 0.005 | 0.003 | 19.650 | 0.059 |  |  | Age | -0.002 | 0.008 | -0.224 | 0.825 |  |
| cfPWV/GLS |  |  |  |  |  | 0.462 | cfPWV/GLS |  |  |  |  |  | 0.154 |
|  | Intercept | -145.390 | 0.444 | -3.272 | 0.003 |  |  | Intercept | 0.300 | 0.645 | 0.466 | 0.647 |  |
|  | AS | -0.039 | 0.062 | -0.625 | 0.537 |  |  | AS | -0.082 | 0.099 | -0.831 | 0.416 |  |
|  | HR | 0.003 | 0.003 | 1.054 | 0.301 |  |  | HR | -0.002 | 0.004 | -0.455 | 0.654 |  |
|  | MAP | 0.012 | 0.003 | 4.036 | <.001 |  |  | MAP | -0.002 | 0.003 | -0.451 | 0.657 |  |
|  | Age | 0.009 | 0.004 | 2.509 | 0.018 |  |  | Age | 0.011 | 0.007 | 1.648 | 0.115 |  |
| baPWV/GLS |  |  |  |  |  | 0.522 | baPWV/GLS |  |  |  |  |  | 0.187 |
|  | Intercept | -126.901 | 51.795 | -24.500 | 0.021 |  |  | Intercept | 113.278 | 98.163 | 1.154 | 0.263 |  |
|  | AS | -0.126 | 7.416 | -0.017 | 0.987 |  |  | AS | -27.392 | 14.924 | -1.835 | 0.082 |  |
|  | HR | -0.406 | 0.313 | -12.956 | 0.206 |  |  | HR | -0.251 | 0.545 | -0.460 | 0.651 |  |
|  | MAP | 1.613 | 0.342 | 47.145 | <.001 |  |  | MAP | -0.222 | 0.498 | -0.446 | 0.660 |  |
|  | Age | 1.148 | 0.433 | 26.499 | 0.013 |  |  | Age | 1.086 | 1.021 | 1.064 | 0.301 |  |
| CAVI/LAR |  |  |  |  |  | 0.209 | CAVI/LAR |  |  |  |  |  | 0.248 |
|  | Intercept | 0.099 | 0.148 | 0.665 | 0.511 |  |  | Intercept | -0.658 | 0.292 | -2.252 | 0.032 |  |
|  | AS | -0.033 | 0.024 | -1.354 | 0.185 |  |  | AS | -0.066 | 0.052 | -1.256 | 0.218 |  |
|  | HR | -1.39e−4 | 0.001 | -0.136 | 0.892 |  |  | HR | -0.004 | 0.002 | -1.796 | 0.082 |  |
|  | MAP | 2.34e-4 | 0.001 | 0.203 | 0.840 |  |  | MAP | 0.002 | 0.002 | 1.145 | 0.261 |  |
|  | Age | 0.003 | 0.001 | 2.366 | 0.024 |  |  | Age | 0.005 | 0.003 | 2.013 | 0.053 |  |
|  | AF | 0.048 | 0.051 | 0.939 | 0.355 |  |  | AF | 0.011 | 0.053 | 0.211 | 0.834 |  |
| cfPWV/LAR |  |  |  |  |  | 0.478 | cfPWV/LAR |  |  |  |  |  | 0.261 |
|  | Intercept | -173.953 | 0.257 | -6.780 | <.001 |  |  | Intercept | -0.967 | 0.322 | -3.006 | 0.005 |  |
|  | AS | -0.074 | 0.042 | -1.760 | 0.088 |  |  | AS | -0.038 | 0.058 | -0.662 | 0.513 |  |
|  | HR | 0.004 | 0.002 | 2.400 | 0.023 |  |  | HR | -0.003 | 0.002 | -1.208 | 0.236 |  |
|  | MAP | 0.005 | 0.002 | 2.460 | 0.020 |  |  | MAP | 0.003 | 0.002 | 1.256 | 0.219 |  |
|  | Age | 0.006 | 0.002 | 3.410 | 0.002 |  |  | Age | 0.007 | 0.003 | 2.323 | 0.027 |  |
|  | AF | 0.140 | 0.088 | 1.600 | 0.120 |  |  | AF | 0.047 | 0.058 | 0.818 | 0.420 |  |
| baPWV/LAR |  |  |  |  |  | 0.400 | baPWV/LAR |  |  |  |  |  | 0.300 |
|  | Intercept | 0.954 | 0.209 | 4.557 | <.001 |  |  | Intercept | 136.922 | 0.283 | 4.841 | <.001 |  |
|  | AS | -0.063 | 0.036 | -1.764 | 0.088 |  |  | AS | -0.078 | 0.051 | -1.536 | 0.135 |  |
|  | HR | 0.001 | 0.002 | 0.751 | 0.459 |  |  | HR | -0.003 | 0.002 | -1.317 | 0.198 |  |
|  | MAP | 0.003 | 0.002 | 1.848 | 0.075 |  |  | MAP | 0.003 | 0.002 | 1.714 | 0.097 |  |
|  | Age | 0.005 | 0.002 | 3.463 | 0.002 |  |  | Age | 0.006 | 0.003 | 2.124 | 0.042 |  |
|  | AF | 0.076 | 0.072 | 1.051 | 0.302 |  |  | AF | 0.013 | 0.051 | 0.246 | 0.807 |  |

**Before AVR** (aortic valve replacement) were considered: baseline heart rate (HR), baseline mean arterial pressure (MAP) and presence of atrial fibrillation (AF). **After AVR** were considered: post-surgery heart rate (HR), post-surgery mean arterial pressure (MAP) and pre-existence or post-surgery occurrence of atrial fibrillation (AF). GLS is considered as absolute value. AS: aortic stenosis indication to surgery; baPWV: brachial-ankle pulse wave velocity; CAVI: cardio-ankle vascular index; cfPWV: carotid-femoral pulse wave velocity; GLS: left ventricular global longitudinal strain; LAR: left atrial reservoir strain;SE: standard error.
